# Demographic and Life-History Responses of *Rhinella arenarum* to Road-Associated Environments

**DOI:** 10.3390/ani15091343

**Published:** 2025-05-06

**Authors:** Mariana Baraquet, Favio Pollo, Manuel Otero, Adolfo Martino, Pablo Grenat

**Affiliations:** 1 Ecología, Departamento de Ciencias Naturales, Facultad de Ciencias Exactas, Físico-Químicas y Naturales, Universidad Nacional de Río Cuarto, Ruta Nacional N° 36–km 601, Río Cuarto X5804BYA, Argentina; mbaraquet@exa.unrc.edu.ar (M.B.); fpollo@exa.unrc.edu.ar (F.P.); motero@exa.unrc.edu.ar (M.O.); amartino@exa.unrc.edu.ar (A.M.); 2 Instituto de Ciencias de la Tierra, Biodiversidad y Ambiente, Universidad Nacional de Río Cuarto—CONICET, Río Cuarto X5804BYA, Argentina

**Keywords:** roads, urbanization, life-history traits, *Rhinella arenarum*

## Abstract

The global decline of amphibians is linked to habitat loss, with urbanization and roads as major threats. Roads fragment habitats and introduce stressors like pollution, temperature changes, and non-native predators. Despite growing awareness, studies in Latin America are scarce. In this study, we examined life-history traits of *Rhinella arenarum* from four sites in central Argentina that differ in urbanization intensity near roads. We analyzed snout–vent length (SVL), age, growth, size at maturity, and longevity in reproductive individuals. Sexual size dimorphism was present across sites, with females consistently larger than males. Moderate variation in SVL and age structure was observed among populations, with smaller and generally younger individuals in sites with medium-to-high urbanization. In contrast, individuals from the least disturbed site tended to show broader age ranges and greater longevity. Growth rates were also slower in the most urbanized site. While differences were moderate and sample sizes limited, these patterns suggest that urban-associated factors may influence demographic traits in *R. arenarum*. These findings contribute to our understanding of how human-altered environments might affect amphibian populations and highlight the need to distinguish between natural geographic variation and anthropogenic impacts on amphibian populations.

## Simple Summary

Amphibians are declining globally, mainly due to habitat loss, with urbanization and roads being significant threats. Roads break up habitats and bring problems like pollution, temperature changes, and non-native predators. Although people are becoming more aware, studies in Latin America are still limited. In this study, we examined how road-adjacent environments with varying degrees of urbanization may influence life-history traits of the common toad (*Rhinella arenarum*) in central Argentina. We analyzed traits such as body size, age, growth, and lifespan at four sites. Females were consistently larger than males, and toads from moderately urbanized sites tended to be smaller and younger. In contrast, individuals from the least disturbed site showed broader age ranges and greater longevity. Growth rates were generally slower in more urbanized areas. While differences among sites were moderate and sample sizes limited, the patterns observed suggest that urban-related factors might influence demographic traits in this species. These findings contribute to the understanding of amphibian responses to habitat disturbance and highlight the importance of further research to disentangle natural variability from anthropogenic effects.

## 1. Introduction

The widely recognized decline of amphibians globally, with more than one-third of the world’s known diversity considered threatened [1,2], has increased interest among scientists to investigate the potential causes of this phenomenon [3]. Among the main threats, habitat loss and destruction are recognized as the number one threat to amphibians worldwide, driven by deforestation and land clearing for agricultural use, urbanization, and industrial development [2,4].

Urbanization is, nowadays, one of the leading widespread causes of alteration and change in natural ecosystems worldwide, making it one of the greatest threats to amphibian populations [4,5,6]. Particularly, the expansion of road networks is a major cause of habitat degradation and fragmentation for wildlife [7,8]. Furthermore, it introduces new stressors, such as non-native predators, exposure to pollutants, warmer temperatures, etc. [9,10].

Over the past few years, there has been a rising focus on the ecological effects of roads on components, processes, and structures at the ecosystem and landscape level [11]. Roads not only represent a structural modification of the landscape but also interfere with amphibians’ ability to move between multiple habitats and recolonize breeding sites [12]. Furthermore, these habitats could be the only ones available and may serve as the only breeding sites for amphibians, though they do not always provide suitable conditions and could even function as ecological traps [13,14]. Numerous studies have demonstrated that roads negatively influence amphibian populations, for instance, by reducing their abundance [15,16,17], assemblage composition [17], endocrine and reproductive functions [18,19], reproductive investment [20], and call properties [21,22,23]. However, the effects of roads on other life-history and demographic traits remain unexplored. Particularly, the age structure of a population affects its growth and provides information about its recent history, current status, and future trends [24]. Therefore, studies are needed to differentiate between natural geographic variation in demographic traits and the impacts of anthropogenic habitat degradation [25]. Amphibian life-history traits frequently vary across populations within the same species [26,27], which is attributed to differences in environmental variables [28,29].

The South American toad *Rhinella arenarum* belongs to the Bufonidae family, is widely distributed across South America, and inhabits a large number of habitat types, including urban areas [1]. Its broad distribution, large population size, and ability to breed in both natural and disturbed habitats make it an especially noticeable species and reinforce its utility as a bioindicator for assessing the impacts of anthropogenic activities on amphibian populations [30]. In particular, it has been well documented for this species that populations exposed to urban and agricultural pressures exhibit significant changes in life-history traits [20,31,32,33,34]. However, the demographic parameters of populations inhabiting environments associated with roads remain understudied.

Despite the growing interest in studying the effects of roads on wildlife, there are few studies on their impact on amphibians in Latin America [35]. In this regard, developed countries have made progress in road network planning and noise mitigation, leading to widespread recognition of their biodiversity impacts. In contrast, these issues remain delayed and insufficiently addressed in less developed countries [17]. In this study, we propose that *Rhinella arenarum* populations inhabiting environments associated with roads are affected in their demographic life-history traits, due to alterations in habitat quality and the introduction of anthropogenic stressors. Therefore, our objective was to assess the effects of roads, with different levels of urbanization, on the life-history characteristics (age and growth, size and age at maturity, and longevity) in reproductive individuals of the common South American toad, *R. arenarum*.

## 2. Materials and Methods

### 2.1. Study Site and Classification

The study area is situated in the periurban area of Río Cuarto city (33°07′23″ S–64°20′52″ W), in the southwest of the province of Córdoba, central Argentina. Located in the Pampa plains of central Argentina, this area is characterized by a gently undulating terrain with a slight slope that facilitates water stagnation. The climate is classified as semi-dry, tending toward semi-humid conditions, with a mean annual temperature of 18 °C (ranging from 23 °C in January to 6 °C in July). Rainfall is seasonal, with the highest precipitation occurring between October and March, averaging between 800 and 1000 mm annually [20,33]. These environmental conditions lead to a period of reduced activity during the colder and drier months, when *Rhinella arenarum* enters a state of overwintering. It is during this time of metabolic slowdown that a single line of arrested growth (LAG) is deposited each year, as has been demonstrated for this species in numerous previous studies [20,32,33,34].

This city is the second largest in the province, strategically located with key road networks linking national and South American commercial corridors. As a result, these roads experience significant vehicular traffic, leading to changes in the surrounding natural or semi-natural environments [17]. Particularly, we selected temporary and permanent wetlands from four study sites, previously characterized by Grenat et al. [17] and Pollo et al. [20]: (1) “NR-A005” is a temporary urban wetland formed by the accumulation of water in flooded ditches on National Road A005 (annual average daily traffic—AADT: high, 10,000 to 15,000 v/d), surrounded on both sides by urban infrastructure, such as residential and commercial areas; this route experiences continuous traffic, mainly during the morning and afternoon, representing the bypass of the city of Río Cuarto, widely used by citizens to move to different points of the city while avoiding crossing it. Furthermore, this route presents an important flow of semi-heavy and heavy traffic due to its strategic commercial location. (2) “NR-36” is a temporary periurban wetland situated on the edge of National Road Nº 36 (AADT: medium-high, +7000 v/d), serving as a connection between two main cities of Cordoba Province: Río Cuarto and Córdoba. (3) “PR-30” is a temporary periurban wetland formed in the ditches of the Provincial Road Nº 30 (AADT: medium to low, with marked increases in summer, 3000–4000 vehicles/day); this road shows increased vehicle traffic only in its initial section near Río Cuarto, primarily due to access to two private residential neighborhoods in the area. (4) “CR” is a permanent wetland formed in an abandoned meander of the Chocancharava River, one of the main watercourses crossing the city, surrounded by the characteristic xeric deciduous forest of the Espinal biogeographic district.

Therefore, water bodies located meters from human settlements and roads were considered because it is known that the extent of the effect of urbanization processes for some species of anuran amphibians is at least 1 km. [12]. Sampling sites were spaced a minimum of 2 km apart to minimize the risk of pseudoreplication. Wetlands were categorized according to the surrounding urbanization. Through the analysis in ArcGIS 10.5-ESRI of high-quality satellite images from Google Earth Pro™ 7.1.8, in a buffer square of 1 × 1 km around each pond, we divided each quadrant into 100 equal cells and counted the number of cells with ≥50% of building and/or road cover. The sites were categorized into four landscape types: high urbanization (≥50%), medium urbanization (≥30 < 50%), low urbanization (≥10 < 30%), and urban open space (<10) [17,20].

### 2.2. Data Collection

Sampling was performed during the reproductive period of the species, from October and December, during the 2017 breeding season. To ensure that all individuals were reproductively active, only pairs in amplexus were sampled. We employed the visual encounter survey technique, which consists of systematically searching along the edges of the wetland to detect couples in amplexus [36]. Individuals were separated and sexed based on secondary sexual traits, including the presence of vocal sacs and nuptial pads, as well as dorsal coloration: males typically exhibit a brownish or greenish back, whereas females display a grayish or light brown dorsum with prominent dark or dark brown blotches [37]. For each individual, we measured the snout–vent length (SVL; mm) using a digital caliper Mahr (0.01 mm, Göttingen, Germany). We also estimated the sexual size dimorphism (SDI): SDI = (size of larger sex/size of smaller sex) ± 1 (+1 if males were larger, −1 if females were larger), and the result was arbitrarily considered positive when females exceeded males in size, and negative when the opposite occurred [38]. The longest toe on the right hind limb of each frog was clipped and preserved in 70% ethanol. To minimize the risk of infection, an antifungal or antibacterial healing agent was applied to the wound site [39]. Following the procedure, individuals were released at their original capture locations. This methodology was approved by the Research Ethics Committee of the National University of Río Cuarto (COEDI-UNRC, registration number 241,242-21).

### 2.3. Age Determination

Skeletochronological studies were carried out in accordance with the protocol proposed by Sinsch et al. [25]: (1) fixation in 4% formol (for at least 12 h), (2) decalcification of bones (10% formic acid, 24 h), (3) paraffin embedding, (4) cross-sectioning of the diaphysis into 10–12 μm slices by a rotary microtome Leica^®^ RM2125 RTS (Leica Biosystems Nussloch GmbH, Nussloch, Germany), (5) staining with Ehrlich’s haematoxylin (2 min), (6) light microscopic count of the number of lines of arrested growth (LAGs), using a light microscope Zeiss Axiophot-Axiolab (40–100X, Carl Zeiss AG, Oberkochen, Germany), and (7) documenting the most informative cross-sections with a digital Axiocam ERc 5s camera (Carl Zeiss Microscopy GmbH, Jena, Germany) and ZEN 2.3 lite 4.3 software.

The age of each individual was estimated following Sinsch [25] for the identification and interpretation of LAGs. A minimum of 10 cross-sections per individual were analyzed, with the number of LAGs counted independently by two observers (M.B. and M.O.) in the periosteal region of the bone. Endosteal resorption was evaluated by identifying the presence of the Kastschenko line (KL), which marks the boundary between the endosteal and periosteal zones [40], and confirmed endosteal resorption following the protocol of [28]. The identification criteria for partial or lightly stained lines, as well as for double lines (considered false LAGs), were detected based on the methodology described by Liao and Lu [41] and Guarino et al. [42].

We estimated life-history traits following Baraquet et al. [43]: (1) age at sexual maturity: minimum number of LAGs counted in breeding individuals; (2) longevity: maximum number of LAGs counted in breeding adults; (3) potential reproductive lifespan: the difference between longevity and age at sexual maturity; (4) SVL at sexual maturity: average SVL of all first breeders with the minimum number of LAGs; and (5) modal lifespan: mode of age distribution.

Growth curves were estimated according to von Bertalanffy’s equation [44] as used in several studies in amphibians [33,43,45,46,47]. This model was fitted to the average growth curve using the least square procedure, and the growth model of body length was regarded as a function of age: SVLt = SVLmax − (SVLmax − SVLmet) e − K (t − tmet), where SVLt = average SVL at age t (mm); SVLmax = maximum asymptotic SVL (mm); SVLmet = average SVL at metamorphosis (mm); t = number of growing seasons experienced (age in years); K = growth coefficient; and tmet = proportion of the growing season until metamorphosis (age at metamorphosis fixed to 0.25 years according to [48]). We considered 11.45 mm as the mean size at metamorphosis according to [33]. Estimates of SVLmax and K with the corresponding 95% confidence intervals were computed using STATGRAPHICS Centurion XVIII (Statpoint, Inc., Warrenton, VA, USA, 2018).

### 2.4. Statistical Analyses

Descriptive data are presented as mean ± standard deviation. The distribution of all measured variables was assessed using the Shapiro–Wilk test for normality and the Levene test for homogeneity of variances. Pearson’s correlation coefficient was used to determine relationships between body size and age. We used ANOVA and Bonferroni post hoc multiple comparisons to compare SVLs between sexes and among sites. For the comparison of age among populations, we used Kruskal–Wallis test and Bonferroni post hoc multiple comparisons. We performed all tests in STATGRAPHICS Centurion XVIII, with α ≤ 0.05 considered significant.

## 3. Results

### 3.1. Body Size and Sexual Variation

Females exhibited a greater average snout–vent length (SVL) than males across all sampled sites (Table 1). The sexual dimorphism index (SDI) was positive in all sites, indicating an intersexual difference in body size, with females larger than males. However, the degree of dimorphism varied among sites: the highest SDI was observed at PR-30 (0.152), followed by NR-A005 (0.080), CR (0.027), and NR-36 (0.021). This variation suggests slight interpopulation differences in sexual size dimorphism, with PR-30 showing the most marked female-biased dimorphism. Pearson correlation tests revealed no noteworthy correlations between SVL and age in females (NR-A005, r = 0.04, *p* = 0.9084; NR-36, r = 0.34, *p* = 0.4525; PR-30, r = 0.09, *p* = 0.8328; CR, r = 0.51, *p* = 0.110) and males (NR-A005, r = 0.05, *p* = 0.873; NR-36, r = 0.45, *p* = 0.309; PR-30, r = 0.25, *p* = 0.547; CR, r = 0.51, *p* = 0.111).

**Figure 1:**
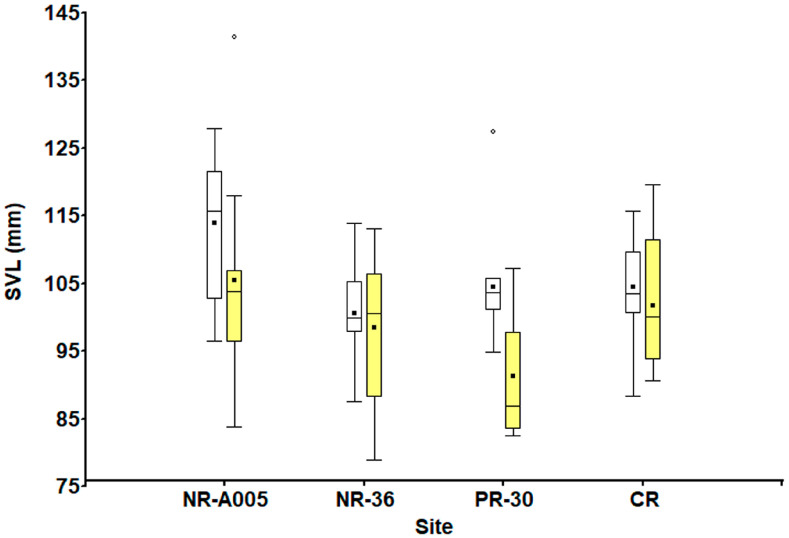
Comparison of SVL (mm) among sites for males and females. White boxes represent females and yellow boxes represent males.

According to the Shapiro–Wilk test (*p* > 0.05), size values for both sexes were normally distributed, and Levene’s test confirmed homogeneity of variance (*p* > 0.05). There was a significant difference in the SVL of females among sites (F_3,80_ = 2.88, *p* < 0.05). Post hoc analyses revealed statistically significant differences between sites, particularly between NR-A005 and NR-36 with each other and with the other sites (Bonferroni test, *p* < 0.05); females at NR-A005 were significantly bigger, while at NR-36, they were significantly smaller (Figure 1). Whereas the SVL of males was statistically different among sites (F_3,38_ = 2.78, *p* = 0.05). In both cases, a higher SVL is observed in individuals from the NR-A005site, with an intermediate SVL at CR and lower SVLs at NR-36 and PR-30 (Figure 1). The SVL at sexual maturity was slightly higher in females than in males at all sites, except at CR (Table 1).

### 3.2. Skeletochronology and Age Analyses

**Figure 2:**
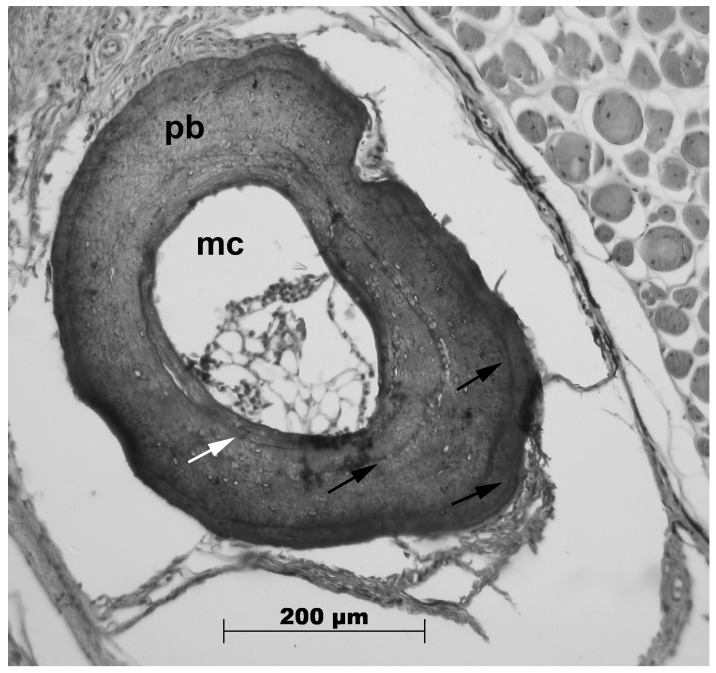
Example of phalangeal cross-section (10 μm thick) of *Rhinella arenarum*. Male, SVL: 96.5 mm; 3 LAGs. Black arrows = lines of arrested growth (LAGs); white arrows = Kastschenko line; mc = medullar cavity; pb = periosteal bone. (Photographed by Manuel Otero).

All adult *R. arenarum* individuals exhibited clearly defined lines of arrested growth (LAGs) in the periosteal bone layer, enabling the estimation of their age (Figure 2). Although endosteal resorption was evident and Kastschenko lines were clearly distinguishable, age estimation was not compromised, as the first LAG was never fully reabsorbed (Figure 2). Double and false lines, defined as incomplete and faintly hematoxylinophilic marks, were observed; however, double lines were counted as a single LAG, while false lines were excluded from the count.

According to the Shapiro–Wilk test (*p* < 0.05), age values for both sexes were not normally distributed, and Levene’s test indicated a lack of homogeneity of variance (*p* < 0.05). Life-history traits of *R. arenarum* showed little variation (Table 1). However, a trend among sites was observed: individuals from high- and medium-urbanization sites differed slightly from those in the urban open space site. The mean age of the females did not significantly differ among the four sites (Kruskal–Wallis test, H = 3.84, *p* = 0.278615). However, mean age significantly differed among sites for males (Kruskal–Wallis test, H = 11.97 *p* < 0.005).

Both sexes exhibited a higher mean age at the NR-36 site, whereas the lowest average age was observed at the PR-30 site (Table 1). Similarly, the highest modal age was registered at all sites for females and at NR-36 and CR sites for males. Greater longevity and reproductive potential were recorded at the CR site for both males and females (Table 1).

### 3.3. Growth Patterns

The von Bertalanffy growth curve correctly fitted the association between age and body length in females across the four studied sites, as revealed by interpopulation comparisons (r^2^ = 96.68 (NR-A005), 98.75 (NR-36), 97.14 (PR-30), 99.27 (CR)), as well as in males (r^2^ = 94.48 (NR-A005), 98.18 (NR-36), 97.89 (PR-30), 98.16 (CR)).

At the PR-30 site, males exhibited a lower asymptotic size (SVLmax) but a higher growth coefficient (K), suggesting that individuals from this population attain their maximum SVL more quickly than those from the other populations. On the other hand, NR-A005 and CR presented coefficients with intermediate values, and the NR-36 site had the lowest coefficient (Table 1). In females, the growth coefficient was higher at the CR site, whereas, SVLmax was higher at NR-A005 than at other sites (Table 1). Nevertheless, there were no significant differences in SVLmax or the growth coefficient among sites, as the 95% confidence intervals for these parameters overlapped across all populations (Table 1).

## 4. Discussion

Natural landscapes are being urbanized on an unprecedented scale around the world. One of the main consequences of this is the proliferation of artificial structures, such as buildings and roads, which constitute one of the greatest recognized threats to amphibian populations [10,35,49]. Therefore, in our study, we aimed at examining potential trait changes in demographic life-history traits of *Rhinella arenarum* in habitats associated with roads. Although our study provides novel demographic insights for four *R. arenarum* populations, the limited sample sizes and only moderate between-site differences suggest that the findings should be interpreted within the context of these constraints.

Our study revealed sexual size dimorphism (SSD), with females larger than males at all four sites, as quantified by the sexual dimorphism index (SDI ranged from 0.021 to 0.152) and in agreement with previous reports on *R. arenarum* [32,33,49,50,51] and other anurans (e.g., [45,52]). However, the magnitude of SSD varied significantly among populations, suggesting that local specific growth patterns, sex specific mortality, and resource availability may differentially shape body size differences [38]. This site dependent variation in SDI highlights the need to consider environmental and demographic contexts when interpreting sexual dimorphism; we explore these potential drivers in the next section.

Numerous studies have shown that anuran body size declines with increasing habitat disturbance [5,53,54,55,56], a pattern we corroborated by finding significantly smaller SVL at the moderately urbanized sites NR-36 and PR-30. Such reductions may reflect a suite of urban-associated stressors, higher solar radiation, lower humidity, and food scarcity, which accelerate larval development and lead to earlier metamorphosis at smaller sizes [57,58,59,60], as demonstrated for *R. arenarum* [34,61,62]. At PR-30, where males exhibited both the smallest mean SVL and the highest sexual dimorphism index (SDI), these developmental shifts appear to impact males more severely than females. If females maintain larger sizes due to fecundity selection, accelerated growth in males under disturbance could amplify female-biased size dimorphism.

Contrarily, toads from the highly urbanized NR-A005 site exhibited the largest SVL. This unexpected pattern may reflect higher prey availability and warmer microclimatic conditions typical of densely urbanized areas [63]. The presence of a major roadway, extensive street lighting, and surrounding commercial infrastructure, such as buildings and paved surfaces, can boost arthropod biomass, the primary prey of *R. arenarum*, thereby enhancing energy intake, digestive efficiency, and growth rates [64,65,66]. Additionally, Pollo et al. [20] reported that populations in similarly urbanized environments maintain reproductive parameters comparable to those in less disturbed areas, highlighting the species’ high phenotypic plasticity and apparent tolerance to urban-associated stressors and potentially leading to modifications in its life-history traits.

In amphibians, body size is generally assumed to increase with age due to their pattern of indeterminate growth. However, age estimation using skeletochronology has revealed that this correlation is often weak or absent, likely due to substantial variation in body size within age classes in many species [58,67,68,69]. Therefore, body size cannot be reliably used as an age predictor, and the relationship between size and age must be analyzed individually for each species [70]. In our study, we found no significant correlation between snout–vent length (SVL) and age in *R. arenarum*. This result is consistent with previous findings in the species [32,33] and may reflect the reduced spacing between lines of arrested growth (LAGs) toward the outer edge of the phalanges, as we observed in most individuals through microscopic examination of bone sections. Moreover, the lack of correlation may be partially explained by the narrow age range detected at most sites, which corresponds to a life stage where growth rates are already declining. As in other amphibians, post-metamorphic growth in *R. arenarum* follows a decelerating trajectory, characterized by rapid early growth that slows substantially after sexual maturity is reached and individuals approach their asymptotic size [33,42,47,48].

Studies such as that of Brady et al. [49], which tested for differences between roadside and woodland populations, unexpectedly reported surprising benefits for amphibian populations breeding and living near roads in terms of size and age. Meanwhile, other studies, like that of Cogălniceanu et al. [55], conducted in permanent freshwater habitats near a medium-traffic road, have suggested that changes in body size and body condition, rather than age parameters, better reflect the response of the common spadefoot toad population to declining habitat quality. However, in our study, largely unfavorable demographic parameters were recorded at NR-A005, NR-36 and PR-30, with PR-30 being particularly notable. The individuals from these sites appear to be comprised of younger and smaller individuals.

Several studies conducted in disturbed environments have reported changes in certain demographic traits of anuran populations, particularly a decrease in mean age [63,64,71], a pattern also observed in *Rhinella arenarum* [33,48,72]. In our study, the average age did not differ between sites in females but varied in males, with breeding adults from CR and NR-36 showing higher average ages in both cases. The modal age, which represents the most common age class in the population, and age range also shed light on the age distribution of *R. arenarum*. The modal age was 4 years at CR (range: 2–6 years) and 4–5 years at NR-36, which explains the higher average age recorded at these sites. In contrast, individuals from roadside habitats with higher urbanization levels, such as PR-30, exhibited lower mean ages and narrower age ranges (3–4 or 3–5 years), which is consistent with previous research linking habitat disturbance to reduced longevity in anurans. A lower average age may reflect increased mortality rates, potentially associated with environmental stressors [71]. In urban and roadside environments, one well-documented source of amphibian mortality is roadkill [73,74], although this has not been specifically assessed in our study area. Such demographic patterns could also contribute to the pronounced female-biased sexual size dimorphism observed at PR-30, possibly reflecting sex-biased mortality. During the breeding season, males are more exposed due to movement and calling behavior, which may increase their vulnerability to predation or collisions, resulting in a younger and smaller male population [75].

Nonetheless, our findings suggest that less disturbed environments, like CR, support a population with a broader age range and greater longevity. This aligns with the evolutionary theory of aging, which proposes that lifespan declines in response to external mortality pressures [76,77]. Moreover, evidence indicates that larger body size enhances survival probability [49], a factor that may be associated with the greater longevity observed in individuals from the CR site.

Some authors have proposed that age at sexual maturity is linked to lifespan, with early maturation often associated with shorter longevity, and delayed maturity more common in species with longer lifespans [78,79]. In our study, individuals from the CR site reach sexual maturity earlier than those from more disturbed sites. In contrast, populations from NR-A005, NR-36, and PR-30 exhibit a higher age at maturity, consistent with the lower growth rates we observed in these three sites, which may reflect slower development under more adverse environmental conditions. The absence of 2-year-old individuals at sites near roads might indicate reduced mating success at this age. As Sinsch et al. [50] noted for *R. arenarum*, individuals with one LAG were often newly matured and unsuccessful in mating, which may explain their absence in our sample because of the sampling method used (couples in amplexus). Furthermore, especially in sites associated with roads, our study confirms that individuals aged 3 and 4 years not only reach reproductive maturity but also successfully participate in mating, indicating that they are actively contributing to the reproductive dynamics of the population. This aligns with evidence that mating success in males is linked to phenotypic traits such as robust forearms, complex calls, and older age [50]. This pattern is more evident near roads with heavier traffic noise, where acoustic pollution disrupts pre-mating behaviors, particularly advertisement calls, reducing the mating success of younger, less competitive males and favoring older, more experienced individuals capable of producing calls that better overcome noise interference [23]. In addition, this dynamic may partly explain the higher sexual size dimorphism observed at PR-30, where larger, older males may have a reproductive advantage under noisy and stressful conditions.

In our study, Von Bertalanffy’s model indicated no significant differences in SVLmax or growth patterns among sites. Although the asymptotic SVL values estimated by the model were larger than those observed, this overestimation may be related to the low representation of small-bodied, sexually mature individuals in our sample [80]. Amphibians generally exhibit indeterminate growth and diverse life-history strategies, involving trade-offs between growth and reproduction that can be influenced by environmental conditions [55,81,82]. In our data, individuals from the most urbanized sites tended to exhibit slower growth rates. This pattern may be associated with environmental stressors common in disturbed habitats, such as chemical pollutants and urban-related stress, which can impair metabolic efficiency and hormonal regulation, ultimately limiting growth [33]. In such conditions, delayed sexual maturity might emerge as a compensatory strategy, allowing individuals to reach the necessary size for reproduction at a later age. This delayed sexual maturity may serve as an adaptive response, allowing individuals to accumulate sufficient energy reserves before reproducing, thus enhancing their reproductive success [83,84].

## 5. Conclusions

In conclusion, assessing the age structure and longevity of populations provides valuable insight into the demographic dynamics of a species and contributes to understanding how environmental factors may influence population stability [85]. In our study, we observed moderate variation in life-history traits across sites with different degrees of urbanization. While our findings are consistent with previous research suggesting that anthropogenic disturbance can affect age-related traits in amphibians, including studies reporting such effects in *Rhinella arenarum* [33,34,48], the small sample sizes and modest differences observed indicate that the results should be interpreted within the context of these limitations. Nonetheless, our results support the view that certain demographic traits, such as age at maturity and growth patterns, may reflect population-level responses to anthropogenic environmental changes. Further studies incorporating larger sample sizes and replicated populations will be necessary to confirm these patterns and clarify the mechanisms driving trait variation in disturbed environments.

## Figures and Tables

**Table 1:** Urbanization categories, SVL (in mm), and life-history traits of *R. arenarum* for each site. Mean ± standard deviation is given for SVL, age, SVLmax, and growth coefficient (K). The range of values is also given in parentheses for SVLmax, and K.

Variable	Males				Females			
	NR-A005 *n* = 12	NR-36 *n* = 7	PR-30 *n* = 9	CR *n* = 11	NR-A005 *n* = 12	NR-36 *n* = 7	PR-30 *n* = 10	CR *n* = 11
Urbanization categories	High	Medium-High	Medium-Low	Urban open space	High	Medium-High	Medium-Low	Urban open space
SVL (mm)	105.44 ± 14.67 (83.87–141.41)	98.38 ± 10.63 (78.88–113.09)	91.26 ± 8.96 (82.45–107.17)	101.77 ± 9.65 (90.66–119.6)	113.94 ± 10.74 (96.56–127.83)	100.53 ± 7.95 (87.5–113.87)	103.52 ± 11.14 (87.25–127.5)	104.53 ± 97.55 (88.38–114.7)
Age (years)	3.67 ± 0.89	4.28 ± 0.48	3.12 ± 0. 35	4.18 ± 0.87	3.90 ± 0.83	4.28 ± 0.75	3.6 ± 0.51	4.18 ± 0.98
Longevity (years)	5	5	4	6	5	5	4	6
Age at sexual maturity (years)	3	4	3	3	3	3	3	2
Potential reproductive lifespan (years)	2	1	1	3	2	2	1	4
Modal age	3	4	3	4	4	4	4	4
SVL at sexual maturity (mm)	105.67	93.77	93.16	93.32	112. 92	99.9	101.8	88.38
SVL_max_ (mm)	109.67 ± 7.16 (94.67–124.66)	100.33 ± 4.69 (90.27–110.4)	97.47 ± 5.52 (83.42–106.84)	108.17 ± 4.36 (99–117.34)	118.5 ± 6.07 (105.75–131.25)	104.68 ± 3.78 (96.58–112.79)	111.15 ± 7.73 (94.66–127.64)	108.31 ± 1.98 (104.15–112.47)
K	0.95 ± 0.37 (0.16–1.73)	0.80 ± 0.22 (0.32–1.28)	0.93 ± 0.26 (0.33–1.05)	0.95 ± 0.16 (0.41–1.09	0.85 ± 0.24 (0.34–1.36)	0.80 ± 0.16 (0.44–1.16)	0.81 ± 0.29 (0.18–1.44)	0.93 ± 0.12 (0.69–1.17)

## Data Availability

All data used for this study are given in the text.
